# Cancer care and COVID-19: tailoring recommendations for the African radiation oncology context

**DOI:** 10.3332/ecancer.2020.1144

**Published:** 2020-11-18

**Authors:** Lotfi Kochbati, Verna Vanderpuye, Rim Moujahed, Mouna Ben Rejeb, Zeineb Naimi, Tajudeen Olasinde

**Affiliations:** 1 Radiotherapy department, Abderrahman Mami Hospital 2090, El Manar University. Tunis, Tunisia; 2 National Center for Radiotherapy and Nuclear Medicine, Korle-Bu Teaching Hospital, Accra, PO Box KB369, Ghana; 3 Ahmadu Bello University / Ahmadu Bello University Teaching Hospital, Shika-Zaria, Nigeria

**Keywords:** pandemic, COVID-19, cancer, radiotherapy, Africa, prioritisation

## Abstract

Africa is the second most populous continent after Asia comprising 54 countries. Given the healthcare system deficiencies in Africa, the impact of the COVID-19 pandemic was expected to be disastrous. The first case of COVID-19 on the continent was reported in Egypt on 14 February 2020. By 13 May, cases had been reported in all 54 countries. Several practice guidelines specific to radiation oncology departments have been published, including prioritisation criteria for postponing radiotherapy, continuation of treatment, hypofractionation or even omitting radiotherapy. The oncology community in Africa has suddenly needed to protect both patients and caregivers and to ensure continuity of essential clinical services despite several challenges. Considering equipment unavailability, lack of human resources and poor infrastructure, tailoring COVID-19 pandemic management to the African context seems mandatory and a unified approach to guideline development in this context is encouraged. In this article, we discuss contextual issues coming into play, highlighting steps to be taken by radiotherapy centres in Africa to mitigate fallouts from the current pandemic to ensure the safety of our patients and staff as well as the impact on future care.

## Background

Africa is the second most populous continent comprising 54 countries after Asia and a mixed bag of economies, the majority being in the low- to the middle-income groups. For decades, health programmes in Africa have been mainly focused on controlling the spread of communicable diseases such as HIV, tuberculosis and malaria. However, cancer is becoming the new ‘epidemic’ in African countries. Available data indicate that more than one million new cancer cases are diagnosed in Africa annually [1]. Radiation therapy is an important therapeutic modality in the management of most cancers. Approximately, 50% of all cancer patients require radiotherapy during the course of their disease [2]. Unfortunately, in Africa, radiotherapy resources are not accessible to a majority of cancer patients. According to the Directory of Radiotherapy Centres database in 2020, only 30 of the African countries are known to have radiation therapy machines with 240 radiotherapy centres recorded, a total of 509 machines out of which most are located in South Africa (129) and Egypt (143), not to mention the huge gap in radiotherapy human resource [3].

Given the surmountable healthcare and social system deficiencies in Africa, the impact of the COVID-19 pandemic was no doubt expected to be disastrous. Reasons for this projection include widespread under-reporting of disease burden in many African countries, low health spending budgets, abounding communicable and noncommunicable disease burden, inadequate sanitation including poor access to running water and socioeconomic practices which preclude social distancing. Africa’s current healthcare systems have inadequate doctor and nurse to patient ratio, suboptimal infrastructure and insufficiently skilled healthcare workers. Specifically, worrying is the reduced capacity for ventilators and intensive care services needed to manage COVID-19 cases and other emergencies during the pandemic. Moreover, healthcare facilities in parts of Africa are frequently subject to shortages of basic supplies such as masks, oxygen, gloves, soap and running water and expected to worsen in the current pandemic.

African states had ample time to prepare and minimize the impact of the COVID-19 pandemic being the last continent to be affected. We learnt from other continents and harnessed our experiences from other epidemics such as the recent Ebola outbreak, resulting in the swift implementation of social distancing, border closures and travel restrictions, school closures, promoting personal hygiene, limiting gatherings and eventually lockdowns. These measures did not exclude clinical patient care, with healthcare services reduced to the barest necessity despite the negative social, economic and cultural implications.

The first case of COVID-19 on the continent was reported in Egypt, North Africa on 14 February 2020 and by 13 May 2020, all 54 African countries had recorded cases. As of 2 October 2020, Africa has recorded 1.5 million cases, 36,400 deaths and 1.2 million recoveries. These reported figures are relatively much lower than expected. However, the average number of test done per 1000 in Africa is low compared to other high-resource regions and could partially account for the lower transmission and case fatality rates seen in Africa [4].

As the virus spreads across the world, there is an increasing concern for the impact of COVID-19 on cancer patients as screening, diagnosis and treatments services are severely disrupted with dire consequences. Moreover, available data have shown a higher susceptibility of cancer patients to COVID-19 with poorer outcomes [5]. The oncology community in Africa has suddenly needed to protect vulnerable patients and caregivers whilst ensuring continuity of essential clinical services. Guidelines for managing cancer during the pandemic have been developed globally by cancer institutions and organisations mostly from well-resourced continents [6]. Notwithstanding, the adaptation of these guidelines necessarily requires tailoring to suit region- or country-specific resources. To date, there are no published data on the incidence and mortality of COVID-19 in cancer patients in Africa.

In this communication, we discuss radiotherapy guidelines in the context of the COVID-19 pandemic and highlight steps taken by radiotherapy centres in Africa to mitigate fallouts from the current pandemic to ensure the safety of our patients and staff even with restrained resources.

## Recommendations during the COVID-19 pandemic: protective measures for radiotherapy staff and patients

The COVID-19 pandemic posed major challenges for African radiation oncology professionals and cancer patients. Several modifications were required in daily practice to prevent the infection spread amongst already vulnerable patients and staff who come in regular direct contact with each other [7]. These measures were adapted based on COVID-19 WHO and international radiotherapy guideline recommendations [6].

Radiotherapy departments implemented measures such as stringent instructions to medical staff and patients about personal hygiene and usage of personal protective equipment (PPE) amongst the clinical staff. All staff and patients, on a daily basis, must visibly wash hands with soap and running water for at least 20 seconds, wear WHO-designated face mask, have their temperature checked and answer pertinent COVID-related clinical questions before entering the facility. Maintaining social distancing of 1–2 m apart, even in the waiting and examination rooms and allowing where unavoidable, one companion per patient into the clinic area is strictly enforced even though admittedly quite challenging due to the regular large patient and family numbers.

In order to prevent cross-contamination, all radiotherapy devices must be thoroughly disinfected following each patient encounter.

The radiotherapy staff were instructed to avoid gatherings; multidisciplinary tumour boards, clinical meetings and educational programs were permitted only via telecommunication. Moreover, staff are scheduled to work on a rotating schedule to avoid cross-contamination among staff, allowing for continuity of care if at any point in time a shift got exposed to the virus.

Symptomatic and suspected patients must be triaged by a specially trained team set up, isolated and referred to the central COVID-19 case management team and should include all staff in direct contact with a suspected case. Treatment is suspended until COVID-19 status is negative.

Being a novel disease, the best strategy to avoid the spread is still unknown, and we all base our hope on the development of a safe and effective vaccine especially for vulnerable cancer patients. Continuing cancer treatment in a cancer patient infected with COVID-19 is considered risky for the patient, staff as well as other patients concurrently receiving treatments within the facility. Radiation therapy may have a negative impact on the immune system, increasing the morbidity and mortality rate for such a patient [7]. The global shortage of PPE supply for health workers was prevalent on the continent. It was not unusual to have disposal garments recycled, further increasing the risk of transmission in high to moderate risk persons. Priority is given to frontline healthcare workers, which usually does not include cancer care workers.

## Prioritisation in the context of Africa

With lessons learnt from high-income countries, radiotherapy units in Africa were prepared to deal with this pandemic despite resource constraints. The common goal of COVID-19 cancer treatment guidelines is to limit patient visits to clinical institutions by balancing the risk of infection and early mortality against the continuation of essential cancer treatments to improve outcomes [6]. This implies adopting cost-effective, less invasive and immunosuppressive therapies. As an example, systemic therapy COVID-19 guidelines limit hospital visits by switching to oral-systemic therapies where applicable [8]. However, the majority of the oral anticancer therapies are disproportionately costly and inaccessible to many parts of Africa exposing the inequalities in global cancer care.

In the field of radiotherapy, COVID-19 guidelines recommend shorter fractionation schedules for palliation and cure where indicated [6]. Shorter fractionation (hypofractionation), especially in the curative setting, mandates the use of advanced and complex skills, equipment for imaging, planning, immobilisation and delivery techniques, required to reduce normal tissue toxicity and maintain the equivalent magnitude of benefit as in longer fractionation schemes. Inadvertently, an adaptation of these guidelines necessarily requires tailoring to suit region- and country-specific resources as many countries in Africa cannot implement these advanced techniques due to existing health system infrastructural gaps. Radiotherapy can be omitted where the therapeutic benefit is judged to be low governed by existing clinical evidence. These choices are not considered suboptimal but for some reason are not popular.

Moreover, treatment decision to omit, delay or modify radiotherapy protocols requires a careful balance of risks, benefits and health-seeking behaviour of the population, especially in the African context with low health literacy, reduced uptake of adequate pain medication and access to other palliative care options. Majority of radiotherapy departments in Africa are faced with a shortage of adequate radiotherapy facilities and skilled human resources resulting in long waiting lists which could be as long as a median of 110 days in a south Africa or 150 days in Ethiopia for curative cases [11, 12]. Large numbers of patients, most of them travelling long distances, are crowded waiting to be admitted into the facility, only a few can be admitted in at a time or cannot be accompanied by family. Long waiting times to be attended to due to the mandatory COVID-19 protocols, limited shift workers and social distancing rules result in disgruntled patients and family and overworked staff. Cancer patients under these circumstances are reluctant to honour scheduled visits, partly compounded by the economic fallout especially in countries with high out of pocket payments and indirect medical expenditure.

A course of curative radiotherapy could take several weeks for its completion. In countries with limited access to radiotherapy facilities as occurs in Africa, many patients have to wait their turn for treatment which could translate in a missed opportunity for improved cancer outcomes. Hypofractionated radiation therapy with shortened overall treatment duration and radiobiological equivalence to conventional fractionation could potentially create the increased capacity to treat additional patients at a lower cost to the patient and healthcare system and improve outcomes. Consequently, less treatment visits minimise the risk of virus transmission by reducing contact with other susceptible patients and reduce burnout on the already limited workforce. Prior to the COVID-19 crisis, arguments promoting hypofractionated regimens in radiation oncology even though supported by evidence were not readily accepted [8]. Lack of real-world data was cited as a barrier to implementation. As an example, hypofractionated radiotherapy schedule for breast cancer using 40Gy/15 fractions instead of 50Gy/25 fractions could treat 1.66 times more patients [13, 14]. Similarly, with FAST (once weekly fractions over five weeks) or FAST FORWARD (five daily fractions over 1 week) regimens, translates into treating five more patients within a 5-week period [15, 16].

Preoperative short-course radiotherapy (I week) for resectable rectal cancer is a recommended treatment of choice over the protracted course (5 weeks) in this period of crisis [17]. Examples of other evidence-based recommendations include prioritisation of single-fraction radiotherapy for bone metastases over longer schedules and 40Gy/15 fractions or even shorter regimen of 25Gy in five fractions in glioblastoma multiforme patients especially in the elderly and/or frail [18-20].

Hormone responsive cancers such as specific categories of breast and prostate cancers could be managed with hormone therapies while radiotherapy is deferred within a reasonable timeframe without affecting prognosis [21]. Hormone therapies require less office visits and are not associated with immunosuppression. Many African countries must, as a matter of urgency, develop resource appropriate protocols for the triaging of radiotherapy based on the expected clinical benefit to guide prioritisation of services. It is not unreasonable to omit radiotherapy and rather implement medical interventions for symptom control when there is evidence of minimal impact on disease control as pertains in patients with less than 3 months of expected survival [9].

Tables 1 and 2 summarise the clinical situation where radiotherapy could be omitted or hypofractionated with the current published evidence supporting the recommendation.

## Challenges of prioritisation in the context of Africa and ethical considerations

The current modelling projections cannot predict the natural history of the pandemic. As the crisis evolves globally, we are facing a new and dynamic medical situation, highlighting the need to modify treatment strategies and develop new paradigms in order to optimize radiotherapy access in the context of our existing health system.

Radiotherapy treatment delay, whenever possible, is strongly advocated during the current pandemic, but raises several issues in the African context. Due to limited resources and high costs of radiotherapy equipment, the majority of our radiation departments have relatively few machines and high workflow and are struggling to treat all referred patients in a timely manner to improve outcomes. In Africa, postponing radiotherapy where chronic long waiting lists already exist may likely create a further unmanageable surge in cancer recurrence in the near future [10]. A high burden of late-stage cancer diagnosis and low healthcare access common to Africa will be exacerbated as less priority is given to cancer control and all resources redirected at controlling the pandemic. Treating patients only when radiation therapy is expected to have a major impact on locoregional control and/or overall survival would be the choice ideal in these circumstances (Table 1). Treatment for curative malignancies such as locally advanced Hodgkin lymphoma, nasopharyngeal carcinoma or cervical carcinoma must be prioritized. We should be mindful of the debilitating psychological impact of withholding or omitting radiotherapy for patients, radiation oncologists and other clinical staff likewise. Many departments with already reduced capacity to fulfil radiotherapy request have to make the decision to prioritize radiation indications and face patients and family care giver’s distress. We encourage in-depth discussions to include the psychologist at virtual multidisciplinary meetings for all patients.

## Lessons learnt

Implementation of hypofractionated radiation schedules and prioritisation of treatment can be considered an effective measure to ensure treatment sustainability during the COVID-19 outbreak and beyond in constrained resource regions including Africa by ‘doing more with less’. Although well-established prospective data for breast, rectal and prostate cancer curative hypofractionated radiotherapy exist, robust evidence for sites such as lung and cervical cancer is still lacking (Table 2).

A collaborative post-COVID evaluation of radiotherapy practices during the pandemic is likely to provide further evidence for the adoption of these strategies.

As the information technology industry rapidly evolves globally and even more so during this pandemic, the application of telemedicine to cancer care is potentially a long-lasting solution to mitigate human resource constraints in Africa. Many African countries suffer critical shortages of qualified medical physicists, therapist and radiation oncologists, and current innovative technologies (remote monitoring, treatment planning, teleconsultation, hands-on teaching and mentoring), and offer cost-effective solutions to manage radiotherapy workflow and enlarge access to expertise. Further investment in safe ethical telehealth approaches with adequate staff training programs are needed to improve radiotherapy access in African countries.

## In summary

Africa has so far controlled the spread of the pandemic despite several coexisting social and economic problems. Cancer patients in Africa and many other resource-constrained regions have suboptimal outcomes for multifactorial reasons including poor screening uptake, late stage at diagnosis, low to access to timely and state-of-art care facilities. The current pandemic and challenging times have exacerbated our already stretched cancer management resources. The additional process required to ensure patient and staff safety place an additional burden on already long waiting times for patients. Several published guidelines to guide the appropriation of radiotherapy applying a prioritisation criterion do not consider resource appropriation and social determinants of health. On the other hand, these new recommendations may have sidelinings that could address improvements in service delivery and outcomes in Africa. This, therefore, underscores the need for radiotherapy centres in Africa to consider the implementation of recommended strategies in the context of pragmatism.

## Conclusion

The current and evolving COVID-19 crisis has resulted in significant changes to radiotherapy practice in Africa. These crucial adjustments will ensure optimal cancer care sustainability and safety for both patients and healthcare givers, emphasising the need to appropriate our limited resources even beyond the pandemic. Tailoring COVID-19 pandemic radiotherapy guidelines to the African context is recommended, and a unified approach to guideline development is warranted.

## Conflicts of interest

The authors declare that they have no conflicts of interest.

## Funding declaration

This manuscript was not supported by funding outside of personal contributions by authors.

## Authors’ contributions

All authors have participated in the study to a sufficient extent to be named as authors. All authors participated in collecting data and writing according to their specialities and participated in the revision of the paper and approved the final manuscript for submission.

## Figures and Tables

**Table 1.** Radiation treatments that may be omitted.

Tumor site	Classification	Treatment intent in classic scenario	Treatment decision in COVID-19 and LMIC context
CNS	-LGG -asymptomatic meningioma G1-2	Adjuvant Curative RT	Omit RT/ salvage RT if recurrence
Oesophagus		Radical CT-RT	Omit RT/ CT alone
gastric		Adjuvant curative RT	Omit RT/ peri-op CT
Lung	Extensive SCLC	Palliative RT PCI	Omit thoracic RT Omit PCI [22]
Pancreatic		palliative	Omit RT/CT alone [23]
Bone metastases	no spinal compression	palliative	Omit RT/BSC

CNS: Central nervous system; LGG: Low grade glioma; SCLC: Small Cell Lung Cancer; IPC: Prophylactic Cranial Irradiation, RT: Radiotherapy; CT: Chemotherapy; BSC: Best supportive care.

**Table 2.** Radiation treatments that may be hypofractionnated.

Tumor site	RT decision	RT regimen (TD/number Fr)	Reference
Breast Elderly/N-	Moderate hypo fraction Short course RT	40 Gy/15 fr 28.5 Gy/5 fr weekly 26 Gy/5 fr daily	START B [14] FAST [15] FAST FARWARD [16]
Glioblastoma Frail/elderly	Moderate hypo fraction Short course	40 Gy/15 fr 25 Gy/5 fr	EORT/TROG [24] IAEA [19]
Rectum	Short course	25 Gy/5 fr	Stokhulm III [25]
Lung Locally advanced	Hypofraction palliative	40 Gy/15 fr 39 Gy/13 fr 16 Gy/2 fr	Canada Norway [25, 26] IAEA [28]
Bone Mets Fracture/spinal compression	Palliative Single fraction	8 Gy/1 fr	P.J. Hoskin *et al* [29] Loblaw DA *et al* [30]
Superior vena cava syndrome	Palliative single fraction	8 Gy/1 fr	IAEA [28, 31]
